# First Robotic Hepatectomy With Middle Hepatic Vein Reconstruction Using ePTFE Graft for Hepatic Adenoma: A Case Report

**DOI:** 10.3389/fsurg.2022.904253

**Published:** 2022-06-14

**Authors:** Jilong Wang, Zongrui Jin, Banghao Xu, Weitao Chen, Jianyong Zhang, Hai Zhu, Tingting Lu, Ling Zhang, Ya Guo, Zhang Wen

**Affiliations:** Department of Hepatobiliary Surgery, the First Affiliated Hospital of Guangxi Medical University, Nanning, China

**Keywords:** case reports, robotic surgical procedures, hepatectomy, hepatic veins, vascular grafting, liver cell adenoma

## Abstract

Surgical resection remains the best choice for the treatment of liver tumors. Hepatectomy combined with artificial vascular reconstruction has been proven as an alternative to treating tumors involving the main hepatic veins. As the cutting-edge surgical technique, robotic liver surgery is a novel procedure expanding the field of minimally invasive approaches, especially in complex reconstruction. This study reports, for the first time, on a robotic hepatectomy with middle hepatic vein (MHV) reconstruction using an expanded polytetrafluoroethylene (ePTFE) graft for a patient with hepatic adenoma. The tumor, which was located in segment 8, was adjacent to the MHV. Robot-assisted resection of segment 4 and partial segment 8, and MHV reconstruction using a ePTFE graft were performed. During the post-operative examination and follow-up, the blood flow of the ePTFE graft was patent, and liver function recovered well. Thus, robotic hepatectomy with MHV reconstruction is a safe, minimally invasive, and precise surgery that may provide a novel approach for patients with liver tumors that are invading or adjacent to the main hepatic veins.

## Introduction

Surgical resection remains the best treatment for liver tumors (1). Hepatic vena cava confluence, vascular involvement of inferior vena cava (IVC), portal vein, and hepatic artery have long been considered contraindications to hepatectomy (2). In recent years, complex hepatectomy combined with vascular reconstruction has become increasingly common due to the development of vascular reconstruction (3). The removed blood vessels can now be replaced with various materials, such as autologous veins, allogeneic blood vessels, and artificial blood vessels. Meanwhile, the development of the robot-assisted hepatectomy technique further promotes the development of precise surgical techniques (4). A robot-assisted hepatectomy with hepatic vein reconstruction has never been reported on. The present study reports, for the first time, on a patient who underwent robot-assisted resection of liver segment 4 (S4) and partial segment 8 (S8) with reconstruction of the middle hepatic vein (MHV) through the successful use of a ringed expanded polytetrafluoroethylene (ePTFE) graft.

## Case Report

### Patient Base Condition and Preoperative Evaluation

**Figure 1:**
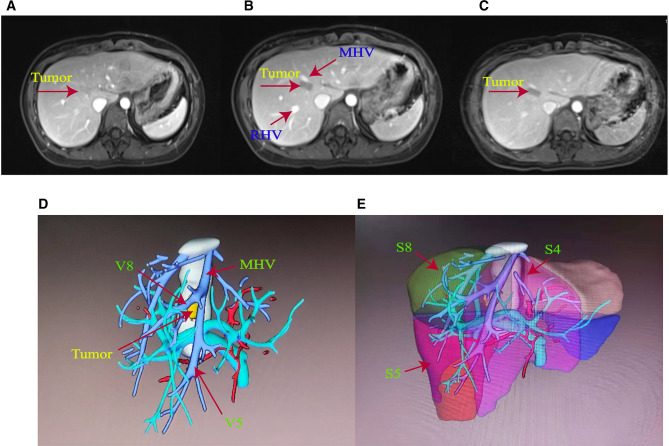
(**A**) Preoperative MRI of the patient with enhanced arterial phase. (**B**) Preoperative MRI of the patient with enhanced venous phase. (**C**) Delayed phase of preoperative MRI enhancement in the patient. (**D**) Main hepatic vein (MHV) trunk and V5 and V8 branches were labeled in accordance with the three-dimensional stereo images of hepatic vessels reconstructed by imaging. (**E**) S4, S5, and S8 reconstruction images of liver segments.

The patient was a 29-year-old female. Routine physical examination and abdominal CT showed a hepatic space-occupying lesion of approximately 1.6 cm × 1.0 cm × 1.3 cm. The patient had a previous history of polycystic ovary syndrome and pituitary microadenomas, and virological examination showed that she did not suffer from liver viruses, such as hepatitis B or hepatitis C. Peripheral blood was biochemically examined for a range of tumor markers, and they were within normal values. Liver MRI plain scan and enhanced examination were further refined, which suggested a lesion in S8 (Figures 1A–C). The lesion in the artery phase showed non-circular high enhancement, the portal vein phase and delay phase were decreased and lower than the liver parenchyma, and the hepatobiliary phase was decreased significantly. The lesion was suspected to be a hepatic adenoma (HCA) or small hepatocarcinoma (HCC).

An IQQA system (EDDA Health Technologies, Inc., Princeton, USA) was used to perform 3D reconstruction. As shown in Figures 1D, E, the tumor was located in S8, near S4 and adjacent to the main trunk of MHV. Liver function was Child–Pugh A, and the liver stiffness score, as examined by the liver elasticity measurement technique, was F1. The retention at 15 min was less than 10% for the indocyanine green test. Liver volume was calculated by the IQQA-3D system. The standard liver volume was 840 ml based on the West China formula (5), and the residual liver volume after resection of S4 and S8 segments was 588 ml, accounting for 70% of the standard liver volume. The reflux areas of the V5 and V8 segments of MHV were 153.7 and 158 ml, respectively.

### Surgical Procedure

**Figure 2:**
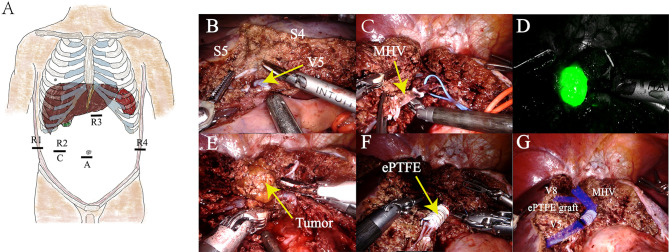
(**A**) Human distribution position of Xi trocar. (**B**) S4 and S5 segments were isolated, revealing the V5 branch of the MHV. (**C**) Trunk of the MHV was cut. (**D**) Intraoperative fluorescence imaging of the tumor was performed to facilitate complete resection of the tumor. (**E**) Isolation and resection of tumors. (**F**) MHV reconstruction using artificial blood vessels of expanded polytetrafluoroethylene material. (**G**) Schematic of the completion of MHV reconstitution.

A da Vinci Xi robot system was used. The patient was positioned in the 12° reverse Trendelenburg position. The procedure was performed with a second surgeon positioned between the patient’s legs. A 12 mm incision was made at the umbilicus, and a trocar was placed for the assistant surgeon. The camera port (8 mm) was inserted in the right abdomen, approximately 10 cm away from the umbilicus. Another three 8 mm ports for the robot were placed in the right hypochondrium, 2 cm below the xiphoid process and left hypochondrium. The first, second, third, and fourth robotic arms were docked on the right hypochondrium, on the camera, 2 cm below the xiphoid process, and on the left hypochondrium ports, respectively (Figure 2A).

The exploration showed pathological adhesion of the upper abdominal bowel and liver cirrhosis on the surface of the liver. No abnormality was found in the gall bladder. Enlarged lymph nodes were found in the first hepatic hilum, with a soft texture. No signs of tumor metastasis were found in the abdominal cavity. Intraoperative ultrasonography revealed a tumor, which was located in S8, adhered with MHV.

The first hepatic hilum was exposed, and a vascular blocking band was preset around the hepatic pedicle. Parenchymal liver transection was achieved using robotic fenestrated bipolar forceps and a harmonic scalpel. The Glissonean pedicles of S4 were ligated and cut. S4 resection was completed. MHV was exposed and stripped, and then, partial segment 8 was resected. As the tumor was close to the V8 branch of MHV, MHV was cut off to completely remove the tumor and avoid MHV bleeding. Following this, the Glissonean pedicles of the tumor were ligated and cut. The tumor was successfully resected under the guidance of intraoperative fluorescence imaging.

The MHV could not be anastomosed directly without tension. An 8-mm-internal ringed ePTFE graft (W.L. Gore & Associates, Inc., Arizona, USA) was used for hepatic vein reconstruction. The MHV was blocked using vascular clamps, and the length of the required ePTFE graft was measured (it was approximately 4 cm). Then, the ePTFE graft was anastomosed to both ends of the MHV using a running suture of 5-0 Prolene. Intermittent irrigation of heparin saline was performed during the anastomoses.

After the reconstruction was completed, the MHV was patent. The operating time was 300 minutes. The intraoperative blood loss was 230 mL. The patient recovered uneventfully and was discharged on the 14th postoperative day. Pathological examination confirmed that the lesion was adenoma, and the subtype was inflammatory. Surgery videos were presented as a supplementary document (Supplementary Video S1 and S2).

### Follow Up

**Figure 3:**
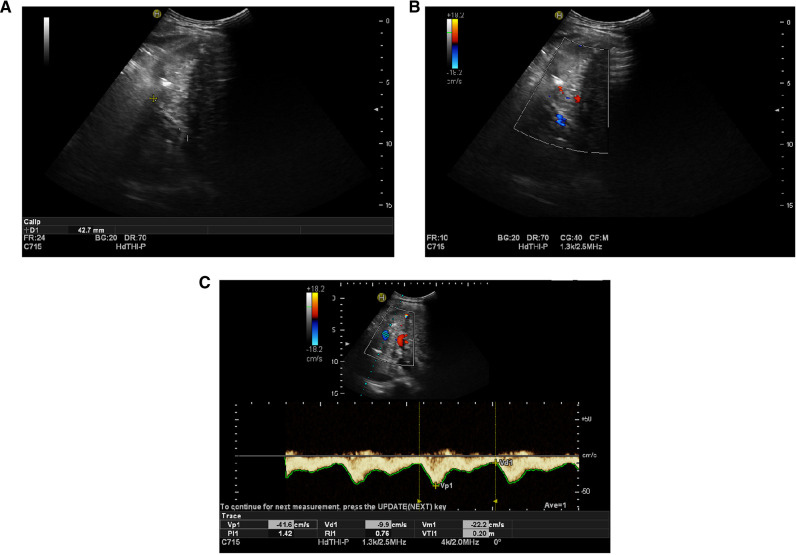
(**A**) Artificial vessels, which were visualized by ultrasound one week after operation, were approximately 42.7 mm in length. (**B**) Blood flow of artificial blood vessels displayed by ultrasound one week after operation (blue). (**C**) Ultrasound blood flow spectrogram of artificial vessels one week after operation, showing blood flow patency.

The patient received rivaroxaban for 3 months after the procedure, and her coagulation profile was checked regularly (Figure 3). Ultrasound blood-flow examinations were performed at 1 week and 3 months after surgery to confirm the patency of the reconstructed vessels. The results showed that the reconstructed MHV was patent.

At the latest follow-up time, which was the ninth postoperative month, the patient’s re-examination showed a good quality of life. The ePTFE graft for the MHV was patent.

## Discussion

Liver resection remains the optimal treatment modality for most liver tumors. Robotic artificial vascular reconstruction combined with hepatectomy can play an important role in the secondary surgery of patients with residual and recurrent tumors, especially when the tumor invades the main hepatic vein or needs to remove the great vein. This fine technique can help these patients avoid liver congestion caused by outflow tract obstruction, which can accelerate the recovery of liver function, improve the quality of life and long-term prognosis (6). Many limitations for liver surgery have been overcome in recent decades thanks to multimodal treatment concepts with staged liver resections, interventional and systemic therapies, and refinement of surgical techniques (7, 8).

Vascular invasion is often considered a contraindication to liver surgery, and such patients often receive only palliative treatment. This is because of the poor prognosis, and the technical difficulties and risks of resection of multiple vessels involved in tumor infiltration. The main goal of tumor surgery is to achieve complete tumor clearance while ensuring patients’ safety (9). The resectability of liver tumors has increased due to the continuous development of new adjuvant treatment modalities and new surgical techniques. Vascular reconstruction combined with hepatic resection is an update in the surgical management of tumors. The progress of techniques and comprehensive treatment has reduced the morbidity and mortality during the perioperative period, and has improved the long-term prognosis of the patients. Nowadays, vascular reconstruction is more frequent (10). Autologous materials are generally the first choice for hepatic vein reconstruction (11, 12). However, the advantage of artificial grafts is the range of length and diameters that can be easily obtained. Therefore, in some cases, artificial grafts have become the ideal substitute. In liver surgery, the artificial grafts mainly used for vascular reconstruction are polyethylene (PTE) grafts and porous polytetrafluoroethylene (PTFE) (13). PTFE has been proven to be a feasible option, with the following advantages over autologous venous grafts: (1) flexibility in complex reconstruction; (2) exact orientation and size calibration for proper fit; and (3) significantly shorter back-table time and anhepatic time (14).

Robot-assisted hepatectomy is becoming increasingly common, and the introduction of robotics in the field of liver surgery may make technically difficult, minimally invasive liver approaches more feasible (15). Robotic liver surgery enables more difficult and refined surgical manipulation through robotic arm advantages, which provide an important basis for complex vascular reconstruction operations. Despite the rapid growth and expansion of robotic liver surgery with promising perioperative outcomes, reports on vascular reconstruction combined with hepatic resection remain limited (16). In several experienced robotic centers, great progress has been made in the manipulation of the hepatic vasculature, and the portal vein, inferior vena cava, and hepatic artery have been safely and successfully reconstructed (17–19). The present paper reports the first case of robot-assisted reconstruction of MHV with artificial blood vessels, combined with a hepatectomy.

HCAs are rare benign tumors of the liver, and the majority (70%–80%) of HCAs are solitary and usually located in the right hepatic lobe (20). At present, according to the molecular biological genotype and pathological characteristics of HCAs, HCAs can be roughly divided into four subtypes: HNF1A inactivated adenoma (H-HCA), β-catenin activated adenoma (β-HCA), inflammatory adenoma, and unclassified adenoma. The inflammatory type is the most common subtype, and β-HCA is the most likely subtype of malignant transformation (21). HCAs are difficult to differentiate clinically from focal nodular hyperplasia and well-differentiated HCC, and the misdiagnosis rate is high. Given that bleeding and malignant transformation are common complications of HCA, surgical treatment is currently advised as early as possible after discovery (22). In the present study, the tumor was close to the junction of the MHV branch and main trunk, and although the volume was small, ablation therapy was not chosen after considering the risk of bleeding and the individual wishes of the patient. Resection of S4 and partial S8 was beneficial to expose the MHV and control hepatic vein hemorrhage, thus providing an adequate perspective to reveal the tumor. Finally, the bleeding volume was decreased.

In liver surgery, maintaining proper blood outflow is as important as maintaining sufficient blood inflow. MHVs are normally responsible for the refluxing of the paracentral segment of the liver, and they have a limited role in the refluxing of S4 (23). Therefore, MHV reconstruction in left hemihepatectomies may be important (24, 25). Reconstruction of the MHV could avoid severe congestion of the refluxing segment, and studies have reported that it could increase the non-hyperemic FLR from 36.5% to 73.8%, which could benefit liver regeneration and avoid the occurrence of postoperative liver failure (26). Severely congested liver segments may cause postoperative liver failure, massive ascites, and impaired liver regeneration (27).

Different criteria for hepatic vein reconstruction in hepatectomies have been published (28, 29). In the authors’ center, the indication for reconstructing the hepatic vein was based on the size (diameter ≥ 5 mm) and depth (superficial or deep) of the hepatic vein. Superficial and deep veins were defined as veins draining 1–2 cm and >2 cm of parenchyma, respectively (30). In the present case, the branch of the MHV (diameter = 7 mm, deep vein) was reconstructed for S5. The MHV could not be anastomosed directly without tension. Thus, an ePTFE graft was used for reconstruction.

Meanwhile, the use of robot technology provided a clear and complete perspective of deep tumor resection and hepatic vein reconstruction. It helps to reconstruct the hepatic vein precisely and prevent the occurrence of postoperative complications related to vascular anastomosis.

## Conclusions

This study reported the first robotic MHV reconstruction combined with a hepatectomy using an ePTFE graft. The patient recovered well, and the reconstructed venous blood flow was patent, which showed that hepatic vein reconstruction combined with a robotic hepatectomy is feasible in an experienced robotic surgery center. The advantages of robotic hepatectomy combined with hepatic vein reconstruction could provide a minimal surgical approach for patients with deep tumors adjacent to large blood vessels.

## Data Availability

The raw data supporting the conclusions of this article will be made available by the authors without undue reservation.
